# Metabolic Regulation of Glycolysis and AMP Activated Protein Kinase Pathways during Black Raspberry-Mediated Oral Cancer Chemoprevention

**DOI:** 10.3390/metabo9070140

**Published:** 2019-07-11

**Authors:** Thomas J. Knobloch, Nathan M. Ryan, Lei Bruschweiler-Li, Cheng Wang, Matthew C. Bernier, Arpad Somogyi, Pearlly S. Yan, Jessica L. Cooperstone, Xiaokui Mo, Rafael P. Brüschweiler, Christopher M. Weghorst, Steve Oghumu

**Affiliations:** 1 Division of Environmental Health Sciences, College of Public Health, The Ohio State University, Columbus, OH 43210, USA; 2 Ohio State University Comprehensive Cancer Center, The Ohio State University, Columbus, OH 43210, USA; 3 Department of Pathology, College of Medicine, The Ohio State University Wexner Medical Center, Columbus, OH 43210, USA; 4 Department of Chemistry and Biochemistry, College of Arts and Sciences, The Ohio State University, Columbus Ohio, Columbus, OH 43210, USA; 5 Campus Chemical Instrument Center, Mass Spectrometry and Proteomics Facility, The Ohio State University, Columbus, OH 43210, USA; 6 Department of Horticulture and Crop Science, College of Food, Agricultural and Environmental Sciences, The Ohio State University, Columbus, OH 43210, USA; 7 Department of Food Science and Technology, College of Food, Agricultural and Environmental Sciences, The Ohio State University, Columbus, OH 43210, USA; 8 Center for Biostatistics, Department of Biomedical Informatics, The Ohio State University, Columbus, OH 43210, USA

**Keywords:** HNSCC, metabolomics, transcriptomics, black raspberries, oral cancer, chemoprevention

## Abstract

Oral cancer is a public health problem with an incidence of almost 50,000 and a mortality of 10,000 each year in the USA alone. Black raspberries (BRBs) have been shown to inhibit oral carcinogenesis in several preclinical models, but our understanding of how BRB phytochemicals affect the metabolic pathways during oral carcinogenesis remains incomplete. We used a well-established rat oral cancer model to determine potential metabolic pathways impacted by BRBs during oral carcinogenesis. F344 rats were exposed to the oral carcinogen 4-nitroquinoline-1-oxide in drinking water for 14 weeks, then regular drinking water for six weeks. Carcinogen exposed rats were fed a 5% or 10% BRB supplemented diet or control diet for six weeks after carcinogen exposure. RNA-Seq transcriptome analysis on rat tongue, and mass spectrometry and NMR metabolomics analysis on rat urine were performed. We tentatively identified 57 differentially or uniquely expressed metabolites and over 662 modulated genes in rats being fed with BRB. Glycolysis and AMPK pathways were modulated during BRB-mediated oral cancer chemoprevention. Glycolytic enzymes *Aldoa*, *Hk2*, *Tpi1*, *Pgam2*, *Pfkl*, and *Pkm2* as well as the PKA-AMPK pathway genes *Prkaa2*, *Pde4a*, *Pde10a*, *Ywhag*, and *Crebbp* were downregulated by BRBs during oral cancer chemoprevention. Furthermore, the glycolysis metabolite glucose-6-phosphate decreased in BRB-administered rats. Our data reveal the novel metabolic pathways modulated by BRB phytochemicals that can be targeted during the chemoprevention of oral cancer.

## 1. Introduction

Oral cancer is estimated to cause almost 50,000 new cases and nearly 10,000 deaths this year in the US alone [1]. Worldwide, it is responsible for 145,000 deaths and is diagnosed approximately 300,000 times yearly, which makes cancers of the oral cavity and pharynx the sixth most common cancer type in the world [2]. Incidence rates for this disease have been increasing steadily every year over the past decade. Despite recent advancements in treatment approaches, the five year survival rate remains at 64.7% [3], and the risk of recurrence among oral cancer survivors remains as high as 36% [4,5]. The majority of oral cancer cases (90%) are considered head and neck squamous cell carcinoma (HNSCC), which affects epithelial cells of the oral cavity, oropharynx, and the laryngopharynx. Major risk factors for oral cancer include tobacco use, consumption of alcohol, and infection with human papilloma virus (HPV) [2]. Given its high prevalence and recurrence rates, it is imperative to identify agents that are able to inhibit the multistep process of oral carcinogenesis, and to define the potential mechanisms of action of these agents.

Black raspberries (BRBs) have been shown to significantly inhibit oral carcinogenesis in both preclinical models and clinical trials [6,7]. Numerous bioactive phytochemicals are contained within BRBs including flavonoids such as anthocyanins, ellagitannins, and phenolic acids [8,9,10]. The complex mixture of phytochemicals present in BRBs has been shown to modulate pro-inflammatory and apoptotic pathways during oral cancer. Furthermore, we showed that BRB mediated oral cancer chemoprevention is associated with a reduction in the expression of cell cycle associated biomarkers [11]. These studies are similar to the effects of BRB during esophageal cancer inhibition [12], where pro-inflammatory pathways were observed to be targets of BRB phytochemicals. The immunomodulatory properties of the BRB phytochemicals cyanidine-3-rutinoside and quercetin-3-rutinoside on immune cells have also been demonstrated in in vitro studies. However, although mechanisms of oral cancer inhibition by BRB phytochemicals are beginning to emerge [13,14,15,16], there remain large gaps in our understanding of how the complex mixture of BRB phytochemicals and their metabolites affect the global metabolic pathways during oral carcinogenesis.

Metabolomics is an emerging technology that identifies and quantifies metabolites that are differentially impacted by alterations in nutrition, environment, and drug administration. Several studies using NMR and MS based metabolomic methods have identified metabolite changes in response to stimuli associated with diseases or treatments [17,18,19]. Targeted and untargeted metabolomic approaches have been developed to track variations in specific metabolites of interest or for a global assessment of metabolic profiles. Data gathered from these approaches can be used to connect metabolite alterations to the modulation of metabolic pathways, leading to the elucidation of potential mechanisms of action [20,21,22]. When combined with transcriptomics, these methods can provide a global picture of potential mechanisms associated with oral carcinogenesis and oral cancer chemoprevention by BRB phytochemicals, and identify novel targets for oral cancer prevention and treatment.

In this study, we used the well-established 4-nitroquinoline-1-oxide (4NQO) carcinogen-induced model of oral carcinogenesis [23] to determine the global metabolic and transcriptional pathways associated with oral cancer chemoprevention by dietary BRBs. Our current study builds upon our previous oral cancer chemoprevention work and expands our knowledge of BRB-mediated oral cancer chemoprevention. Our innovative integrated multi-omics approach identified genes and metabolites modulated in vivo after BRB consumption during experimental oral carcinogenesis, and uncovered novel pathways associated with oral cancer chemoprevention by a BRB-supplemented diet including pathways associated with MAP kinase, carbohydrate metabolism, glycolysis, protein kinase, DNA repair, and cell cycle checkpoint regulation. Each of the pathways identified present novel targets for oral cancer chemoprevention.

## 2. Results

### 2.1. BRBs Modulate the Metabolic Profile of Oral Cancer Induced Rats

We previously showed that BRBs inhibited oral carcinogenesis in rats exposed to the carcinogen 4NQO [11]. In this model, oral lesion incidence and multiplicity were significantly reduced by 5% and 10% BRB-supplemented diet administration following 4NQO exposure when compared to non-BRB treated 4NQO exposed rats. This association between BRB consumption and a reduction in oral lesions corroborates other studies demonstrating the anti-cancer properties of dietary BRBs [24].

**Figure 1:**
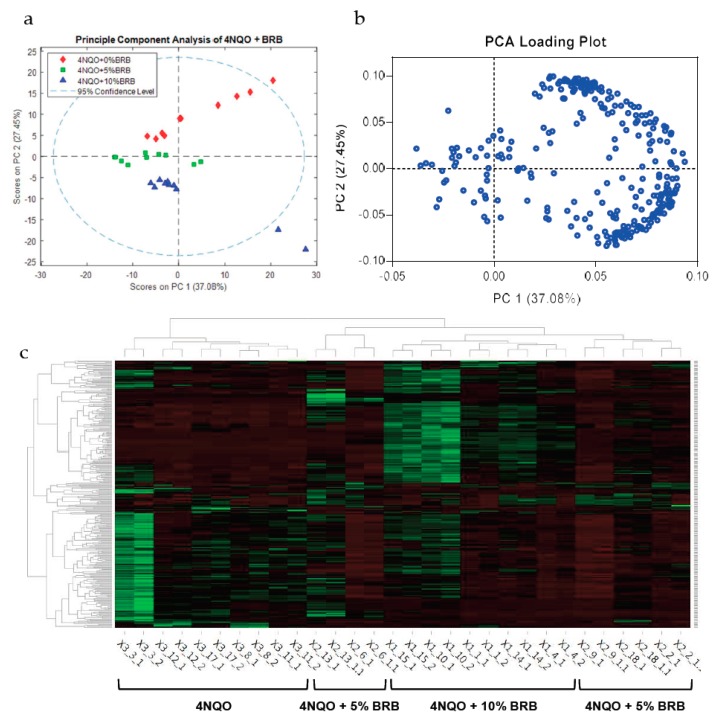
BRBs modulate the metabolic profile of oral cancer induced rats. (**a**) PCA of untargeted metabolomic profiles of urine samples from cancer-induced rats fed the control diet, 5% BRB diet, or 10% BRB diet as determined by LC-MS using the 292 features below the 0.05 *p*-value cutoff (ANOVA). (**b**) Loading scores for PC1 and PC2 for the top 292 features used in the PCA in (**a**). (**c**) Heat map of differentially expressed metabolites in 4NQO-exposed rats fed the control diet (4NQO), 5% BRB supplemented diet (4NQO + 5% BRB), or 10% BRB supplemented diet (4NQO + 10% BRB).

A comprehensive analysis of metabolites in urine samples from carcinogen-induced rats with or without BRB intervention using an LTQ Orbitrap XL mass spectrometer demonstrated clear clustering between groups by unsupervised PCA analysis. Clustered groups include 4NQO-exposed rats not receiving dietary BRBs, 4NQO-exposed rats fed a 5% BRB diet, and 4NQO exposed rats fed a 10% BRB diet (Figure 1a,b).

LC-MS analysis of urine samples from 4NQO-exposed rats administered with BRB-supplemented or the control diet revealed a distinct expression of metabolites in 4NQO-exposed rats fed 5% or 10% BRBs when compared to 4NQO-exposed rats fed the control diet (Figure 1c). Metabolic pathway analysis of differentially expressed metabolites from our LC-MS data using MetaboAnalyst showed that metabolic pathways associated with carbohydrate metabolism are predicted to be modulated by BRBs during the chemoprevention of oral carcinogenesis. The predicted pathway analysis profiles modulated in the BRB fed, 4NQO exposed rat groups are summarized in Table 1.

We also performed an in-depth analysis of the metabolites in urine samples from representative rats from each group using ‘Complex Mixture Analysis by NMR’ (COLMAR) followed by 2D ^13^C-^1^H HSQC NMR quantitative analysis. We identified 123 metabolites in the urine samples of 4NQO-only exposed rats while 126 and 115 metabolites were identified in 4NQO exposed rats that were fed 10% and 5% BRB, respectively. There was one unique metabolite identified in 4NQO-only exposed rat urine samples that was absent in the BRB administered groups. There were seven unique metabolites identified in the BRB administered groups that were absent in the 4NQO-only exposed rat urine samples.

Eleven other metabolites had increased concentrations and ten metabolites had decreased concentrations following BRB administration of the 4NQO exposed rats when compared to rats exposed only to 4NQO. These data are summarized in Table 2.

These data suggest that BRBs downregulate and upregulate metabolic pathways associated with 4NQO-induced oral carcinogenesis, and provide insights into distinct metabolic markers and pathways that may drive BRB-mediated oral cancer chemoprevention.

### 2.2. Modulation of Global Transcriptional Profiles during Oral Cancer Chemoprevention by BRBs

**Figure 2:**
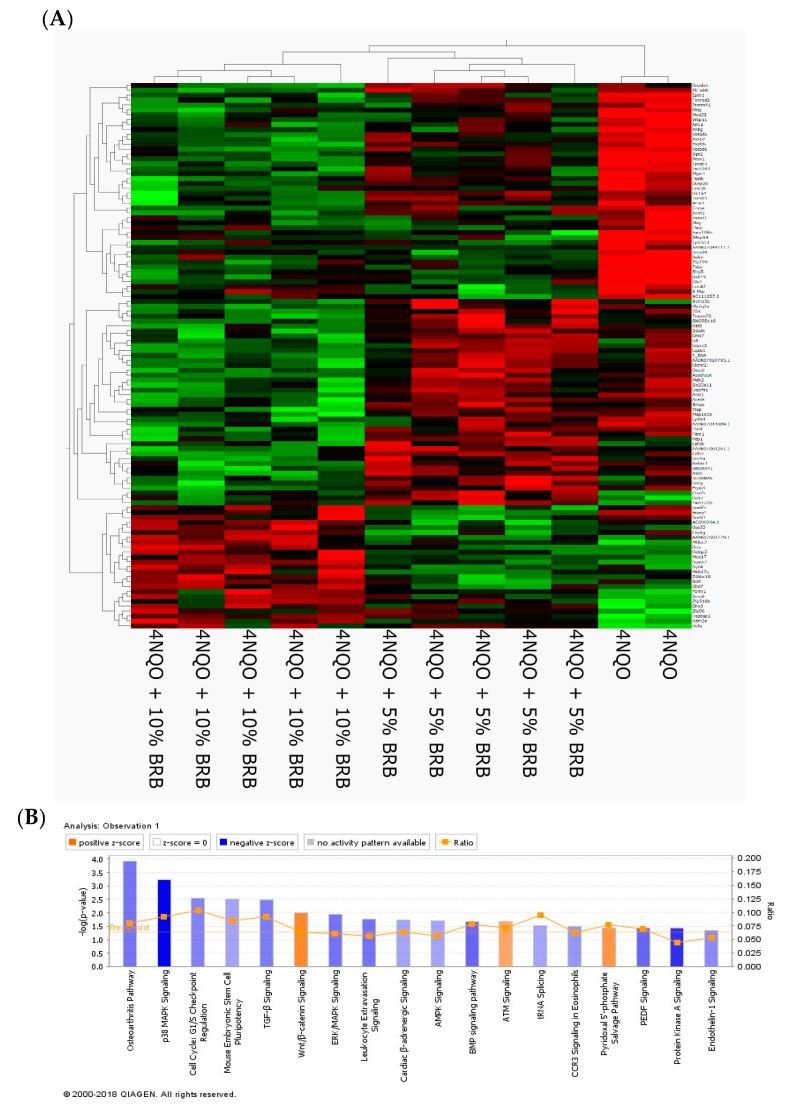
Modulation of global transcriptional profiles during oral cancer chemoprevention by BRBs (**A**) Heat map showing differentially expressed genes from rat tongues during 4NQO induced oral carcinogenesis and BRB mediated oral cancer chemoprevention. 4NQO exposed rats were fed a control diet, 5% BRB, or 10% BRB supplemented diet. (**B**) Ingenuity pathway analysis of the canonical transcriptional pathways modulated by dietary BRB intervention during oral carcinogenesis. Transformed RNA sequencing data from rat tongue RNA from 4NQO exposed, BRB treated, and untreated rat groups were analyzed using Ingenuity Pathway Analysis software (Qiagen).

We analyzed the total gene expression using RNA-Seq of rat tongue tissue from BRB fed and 4NQO-only exposed rats. We identified 662 genes that were differentially expressed among the 4NQO, 4NQO + 5% BRB, and 4NQO + 10% BRB groups (Figure 2A). Detailed analysis of our transcriptional data using ingenuity pathway analysis (IPA) revealed a network of associated pathways that are linked to oral carcinogenesis and BRB-associated chemoprevention. Importantly, similar or overlapping pathways were identified in both the 5% and 10% BRB fed groups when compared to the 4NQO-only exposure group, supporting a consistent pattern of transcriptional modulation by BRB phytochemicals. These oral cancer-associated pathways included genes involved in cancer development, cell cycle regulation, MAP kinase signaling, leukocyte extravasation, and DNA repair, which were regulated in a manner that supports a cancer preventive role for BRB during oral carcinogenesis (Figure 2B).

To confirm the differentially expressed genes identified by RNA-Seq, we performed quantitative RT-PCR (RT-qPCR) validation on a larger cohort of rat tongue RNA samples from each group. The genes analyzed represent the entire range of BRB-mediated chemopreventive pathways we identified through the analysis of our RNA-Seq data. Our results demonstrated that multiple pathways are involved in BRB-mediated oral cancer chemoprevention, which can potentially be targeted for intervention strategies and adjuvant treatment of oral cancer. Significant molecular and metabolic pathways modulated by BRB during oral cancer chemoprevention are discussed below.

### 2.3. Modulation of the Glycolytic Pathway by BRB during Experimental Oral Carcinogenesis

**Figure 3:**
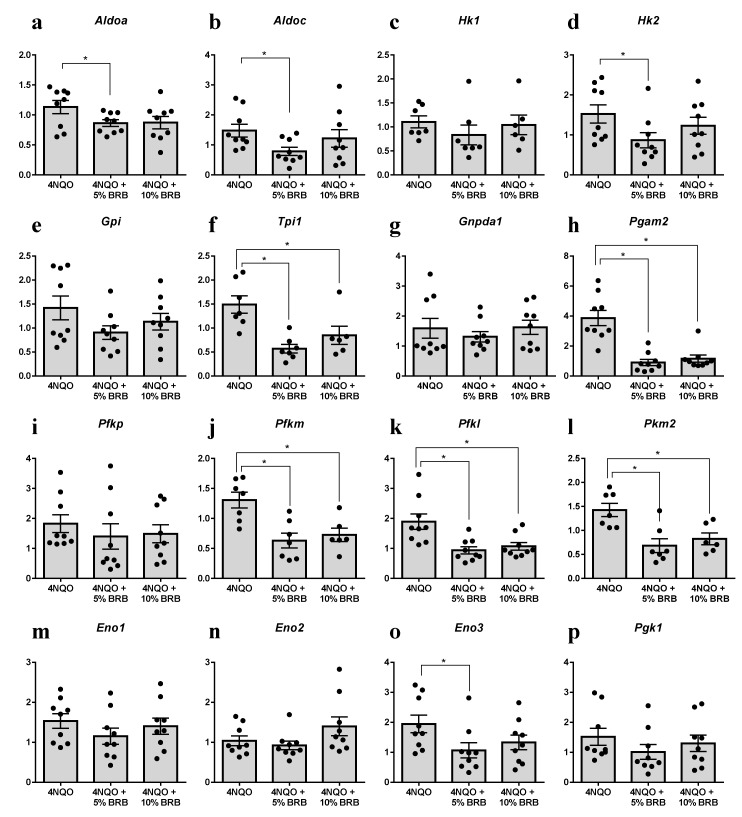
Modulation of the glycolytic pathway by dietary BRB during oral carcinogenesis. (**a**–**p**) gene expression profiles of the glycolysis pathway genes in rat tongue RNA from 4NQO-exposed rats fed the control diet (4NQO), 5% BRB supplemented diet (4NQO + 5% BRB), or 10% BRB supplemented diet (4NQO + 10% BRB), as determined by RT-qPCR. (* *p* < 0.05).

**Figure 4:**
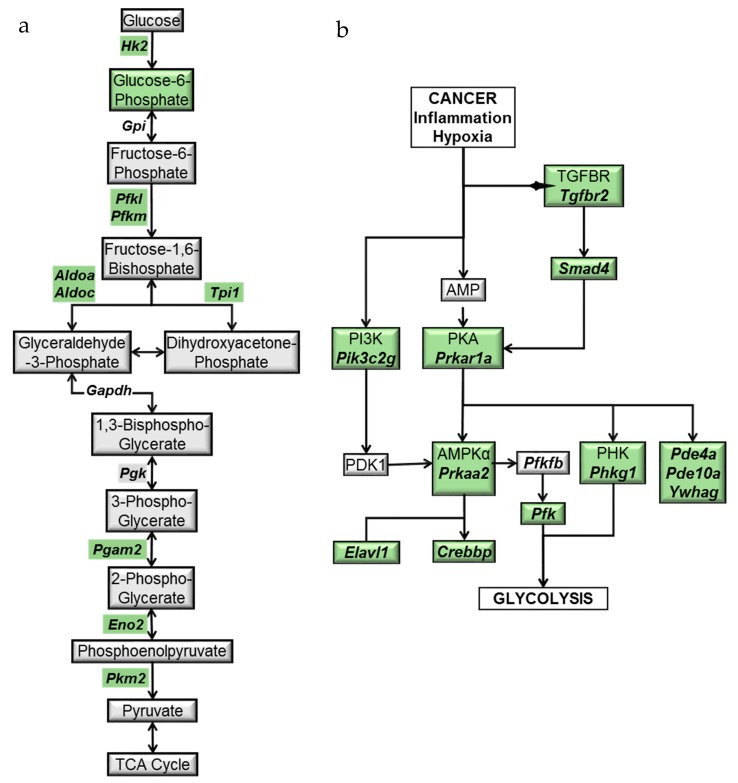
Model of the (**a**) glycolysis and (**b**) PKA-AMPK pathway genes and metabolites modulated by dietary BRBs during oral carcinogenesis. Downregulated genes and metabolites are shown in green.

An integrated analysis of transcriptomic and metabolomic data by IPA and MetaboAnalyst revealed a significant modulation of genes and metabolites associated with the glycolytic pathway (Figure 3 and Table 1). This is significant because enhanced glucose fermentation even in the presence of oxygen and functioning mitochondria, known as the Warburg effect, is a major metabolic mechanism used by cancer cells to promote proliferation and survival, and targets of this pathway are being developed for cancer therapy [25,26]. Our data showed that Aldolase A (*Aldoa*), a glycolytic enzyme, was significantly downregulated in the oral tissues of the BRB administered 4NQO-induced rats when compared to 4NQO-only cancer-induced rats. Similarly, NMR and LC-MS metabolomic analysis showed a significant reduction in glucose-6-phosphate in the 4NQO-exposed rats fed a 5% or 10% BRB supplemented diet when compared to the 4NQO-exposed rats fed the normal diet (Table 2). Based on the results of our RNA-Seq gene expression data, we performed further transcriptional analysis of genes associated with the glycolytic pathways on a larger cohort of rat tongue RNA samples from 4NQO-exposed rats fed with a normal or BRB supplemented diet by RT-qPCR. The glycolytic enzymes *Aldoa*, *Aldoc*, hexokinase 2 (*Hk2*), triose phosphate isomerase 1 (*Tpi1*), phosphoglycerate mutase 2 (*Pgam2*), phosphofructokinases (*Pfkm* and *Pfk1*), pyruvate kinase, muscle (*Pkm2*), and Enolase 3 (*Eno3*) were downregulated by 5% and/or 10% BRB administration in the 4NQO exposed rats (Figure 3). Our data demonstrated that the perturbation of metabolic pathways associated with glucose metabolism are key mechanisms of oral cancer inhibition by BRBs. Taken together, our results suggest that enzymes in the glycolytic metabolic pathway are potential targets for oral cancer chemoprevention by BRBs (Figure 4a).

### 2.4. BRB Downregulates Protein Kinase A and AMP-Activated Protein Kinase Signaling Pathways

Given that glucose metabolism is partly regulated by the serine threonine protein kinase AMP-activated protein kinase (AMPK) [27], we next investigated the impact of BRB administration on the signaling pathways associated with this essential metabolic regulator as well as the related protein kinase A (PKA). This was of particular interest because under certain metabolic stresses including glucose starvation, AMPK reroutes metabolic processes to allow for alternate cellular survival and proliferation strategies. Our data demonstrated that BRB modulates this central metabolic regulator. Dietary administration of 5% and 10% BRB downregulated the expression of essential genes associated with the AMPK pathway. Importantly, the expression of the protein kinase AMP-activated catalytic subunit alpha 2 (*Prkaa2*), which encodes the AMPKα subunit, was downregulated by 5% and 10% BRB administration (Table 3). Similarly, downstream targets of the AMPK pathway including the RNA binding protein (*Elavl1*), CREB binding protein (*Crebbp*), and the stimulatory G-protein alpha subunit (*Gnas*), known to be elevated in various cancers, were downregulated by BRB administration. 

Next, we analyzed the expression of an associated metabolic pathway and upstream regulator of AMPK signaling, protein kinase A (PKA), which is also dependent upon cAMP phosphorylation. In its active form, PKA is able to activate transcription factor NF-κB, resulting in an upregulation of many genes associated with oncogenesis. Our data showed that, similar to AMPK signaling, the administration of 5% or 10% BRB inhibited the PKA signaling pathway in oral cancer induced rats. Targets of the PKA complex such as the phosphodiestarases (*Pde4a* and *Pde10a*) were downregulated by 5% and 10% BRB administration during oral carcinogenesis (Table 3). Furthermore, a reduction in the expression of target genes shared in common between the PKA and AMPK metabolic pathways suggests a mechanism of BRB oral cancer chemoprevention that is mediated by the PKA–AMPK axis (Figure 4b).

## 3. Discussion

Our metabolomic and transcriptomic data revealed a number of novel metabolic pathways modulated by BRB as well as the altered transcriptional profile associated with these modulated pathways as a result of BRB-mediated chemoprevention during oral carcinogenesis. BRBs comprise a complex mixture of phytochemicals, and we are continuing to identify the distinct bioactives that drive the oral cancer chemoprevention pathways observed in the current study. Nevertheless, our data provide progressive evidence for the modulation of pathways associated with established and emerging hallmarks of cancer including deregulated metabolic pathways, inflammation, cellular invasion, and metastasis [28], which present themselves as potential therapeutic targets for HNSCC treatment.

Surrogate systemic biomarkers of local efficacy as well as mechanistic indicators of systemic efficacy are ways to monitor carcinogenesis and chemopreventive impact. Urine, serum or plasma, and saliva are standards for the assessment of biosystematic distribution and availability. In this study, we were interested not only in the locoregional metabolic landscape of the epithelial tumor environment, but also the broader role of systemic mediators of carcinogenesis and cancer chemoprevention. We have previously used both localized site-specific tongue tissue as well as systemic mediators of impact to better define the tumorigenic landscape [13,29]. Our results highlight the necessity of examining both the localized and systemic mechanisms of carcinogenesis and prevention.

The Warburg effect is a known survival mechanism employed by cancer cells. Requiring large amounts of energy for rapid and limitless division, cancer cells are known to hijack the metabolic pathways of the host and upregulate glucose uptake and aerobic glycolysis to support their large energy demands [30]. This results in an accumulation of intermediate glycolytic metabolites in the tumor microenvironment, which provide essential anabolic support (nucleotide, amino acid, and lipid biosynthesis) for the proliferation of cancer cells and subsequent tumor growth. Inhibition of the glycolytic metabolic pathway, and therefore the reversal of the Warburg effect, has long been a target in the prevention of oral cancer [31]. In practice, however, HNSCC cells demonstrate a high propensity to modulate metabolic pathways, rendering inhibition of the glycolytic pathway ineffective due to the cancer cell’s ability to circumvent the inhibited glycolytic process [32,33]. Despite this, our data suggest that a potential mechanism of oral cancer inhibition by the complex mixture of BRB phytochemicals is an inhibition of the glycolytic pathway. Although the specific phytochemicals that drive this metabolic regulation and the detailed signaling cues that cause the inhibition of glycolysis during BRB-mediated oral chemoprevention are yet to be fully elucidated, our results suggest that *Aldoa* was targeted by BRB. Although the role of *Aldoa* in oral cancer is incompletely understood, this key glycolytic enzyme has been shown to be highly expressed in squamous cell carcinoma of the lung, and correlates with tumor metastasis and poor prognosis [34]. Furthermore, depletion of *Aldoa* expression in lung squamous carcinoma cells reduces tumorigenesis [34]. Similar roles for *Aldoa* in tumor progression and metastasis were observed in hepatocellular carcinoma [35], pancreatic cancer [36], and colorectal cancer [37]. Indeed, this glycolytic enzyme is becoming increasingly recognized as an emerging drug target in cancer chemoprevention [38]. As demonstrated by our results, targeting this enzyme is likely to be a potentially novel approach to oral cancer chemoprevention and therapy. Interestingly, *Hk2*, which is known to be highly expressed in many cancers [31,39,40,41,42,43,44], promotes tumor initiation and oncogenic transformation and was selectively inhibited by BRB during experimental oral carcinogenesis. Similarly, the glycolytic enzymes *Pkm2*, *Tpi1*, *Pgam2*, and *Pfkl* are known to be highly expressed in various cancers, and are associated with poor prognosis [39,45,46,47,48,49,50,51,52,53,54,55]. It was therefore not surprising to observe that experimental oral cancer inhibition by dietary BRBs was associated with a downregulation of these glycolytic enzymes by BRBs. Although the precise mechanisms underlying the BRB-mediated targeting of key enzymes of the glycolytic pathway need to be fully clarified, our data provide evidence that a novel mechanism of oral cancer chemoprevention by BRB phytochemicals is the metabolic regulation of glycolysis.

Our data also revealed other metabolic pathways closely associated with glycolysis that are modulated by BRB phytochemicals during experimental oral carcinogenesis, which potentially contribute to the reduction of oral lesions. One such associated pathway is the AMPK pathway, which is an important central regulator of glucose metabolism in response to metabolic stress [56]. Although AMPK has been described as a tumor suppressor in certain cancers by inhibiting cancer cell proliferation [57], it has been shown to promote tumor growth in the context of some breast and epithelial cancer models by promoting tumor cell survival [58,59]. It is therefore evident that the role of AMPK in cancer is context dependent [60,61,62]. Indeed, in the context of metabolic stress, the role of AMPK in maintaining energy homeostasis by suppressing anabolism and promoting catabolism as well as neutralizing reactive oxygen species appears to be tumor protective [60,61,62]. These latter studies corroborate our findings which suggest a downregulation of the AMPK metabolic pathway by BRB as a mechanism of oral cancer chemoprevention.

Interestingly, recent studies have shown that under certain circumstances, overexpression or activation of AMPK is a mechanism by which oral cancer cells are able to survive molecular targeted therapy [63]. This was observed in the case of the cetuximab treatment of several HNSCC cell lines [63] where the activation of AMPK promoted cancer cell resistance to cetuximab. It therefore follows that chemopreventive approaches that inhibit AMPK activity can potentially improve the efficacy of oral cancer treatment by cetuximab. The fact that BRBs inhibited AMPK expression in our experimental oral carcinogenesis model is supportive of a role for BRB as a complementary chemopreventive agent in combination with molecular targeted therapies like cetuximab in oral cancer treatment. A recent study demonstrated the therapeutic potential of targeting both glycolysis and AMPK in cancer therapy. It was shown that the combined inhibition of glycolysis and AMPK synergistically enhanced cytotoxicity of breast cancer cells but not normal cells [64]. In this study, inhibition of glycolysis by 2-deoxyglucose resulted in AMPK activation due to decreased ATP levels, and this recovery of cellular ATP counteracted the cytotoxicity of the glycolytic inhibitor. Co-administration of an AMPK inhibitor inhibited cellular ATP recovery and enhanced the cancer cell killing effect of 2-deoxyglucose [64]. It is therefore not surprising that the combined inhibition of the AMPK and glycolysis pathway by BRB phytochemicals improves its chemopreventive efficacy during experimental oral carcinogenesis. Furthermore, it demonstrates the viability of BRB phytochemicals in targeting metabolic pathways during oral cancer chemoprevention.

In addition to targeting metabolic pathways, integrated analysis of our multi-omic data further confirmed the array of molecular pathways modulated by BRB phytochemicals. For example, previous studies by ourselves and others demonstrate modulation of the pro-inflammatory transcription factor NF-κB by BRB phytochemicals during oral carcinogenesis [11,65], and our current data corroborate and extend this observation. Interestingly, we further showed that a possible mechanism of NF-κB modulation by BRB is through the PKA pathway. PKA associated NF-κB activation has been directly linked to a poor prognosis in oral cancer, and inhibition of PKA was shown to inhibit tumor cell proliferation, induce cell death, and modulate a number of pro-inflammatory and angiogenic genes associated with NF-κB activation [66,67]. PKA has also been shown to play a role in the suppression of anti-tumor immune surveillance of the host [68], making it a particularly attractive target for chemoprevention. Previous studies have demonstrated inhibition of NF-κB by BRB phytochemicals and this inhibition is associated with a reduction in oral carcinogenesis [6,7,11,69]. It is possible that BRB inhibits NF-κB phosphorylation by interfering with PKA signaling. Furthermore, genes involved in the PKA pathway shown to be inhibited by BRB during experimental oral carcinogenesis (*Crebbp*, *Gnas*, and *Ywhag*) have been identified as possible targets for the treatment of HNSCC, breast, and non-small cell lung cancer [70,71,72]. Our results provide a plausible link between the attenuation of the PKA signaling pathway and the previously reported reduction in NF-κB mediated inflammation and cellular proliferation during oral cancer chemoprevention by BRBs [6,11].

In conclusion, by using an integrated approach that combines metabolomic and transcriptomic analysis, we showed that the modulation of metabolic pathways associated with glycolysis and AMPK signaling represent novel targets for natural product interventions with BRBs during oral carcinogenesis. Additional studies will be required to fully characterize the exact phytochemicals in BRB that target these pathways, and the key mechanisms underlying the phytochemical mediated modulation of these essential metabolic pathways during oral carcinogenesis. However, it is likely that multiple bioactive phytochemical compounds or phytochemical groups in BRB are involved in these mechanisms, and it is the innate combinatorial interaction of these phytochemicals that is required for optimal oral chemopreventive efficacy. The application of metabolomic methods to identify and characterize BRB metabolites during oral cancer chemoprevention will continue to provide additional insights into strategies to exploit the bioactive phytochemicals in BRB for cancer prevention and treatment.

## 4. Materials and Methods

### 4.1. Animals

Male F344 rats were housed in a facility at the Ohio State University according to animal protocols and under regulation of the University Laboratory Animal Resources (ULAR). All animal experiments were performed with the approval of the Ohio State University Institutional Animal Care and Use Committee (Protocol #2010A00000085) as well as the Institutional Biosafety Committee.

### 4.2. Chemicals

The chemical carcinogen 4-nitroquinoline-1-oxide (4NQO) was purchased from Sigma-Aldrich (St. Louis, MO, USA). Stock aliquots of 4NQO were weighted en masse and stored at −20 °C in foil wrapped containers. Fresh working solutions of 20 μg/mL 4NQO in drinking water were prepared weekly. BRBs (*Rubus occidentalis* Jewel variety) were purchased from the Stokes Berry Farm (Wilmington, OH, USA), cleaned, and frozen on-site. Whole frozen BRBs were shipped to Van Drunen Farms (Momence, IL, USA) for freeze drying and pulverization, generating BRB powder. BRB powder was stored at −20 °C before incorporation into custom AIN-76A animal diet pellets (Dyets, Inc.; Bethlehem, PA, USA) at both 5% and 10% w/w concentrations as previously described [11].

### 4.3. Experimental Oral Carcinogenesis

Oral carcinogenesis was induced in male F344 rats (6–7 weeks) as described previously [11]. Briefly, rats were randomized into a sentinel group (Group 1, *N* = 20) and three experimental groups (Groups 2–4, *N* ≥ 30 per group). Experimental groups were exposed to drinking water containing 20 μg/mL 4NQO for 14 weeks. At week 14, 4NQO drinking water exposure in the experimental groups was terminated, and all groups were provided with standard drinking water for an additional six weeks. The sentinel control group (Group 1) received standard drinking water without any 4NQO carcinogen exposure and was fed control the AIN-76A diet for the duration of the study. Group 2 was exposed to 4NQO in drinking water and received an unmodified AIN-76A diet for the full 20-week duration of the experiment. Groups 3 and 4 received the AIN-76A diet supplemented with 5% and 10% w/w BRB, respectively, for the six-week period following the termination of 4NQO exposure. Urine was collected at week 20 for metabolomic analysis through complex mixture analysis by NMR (COLMAR), which is based on 2D ^13^C-^1^H HSQC NMR spectra and Orbitrap mass spectrometry. Rat tongues were resected and stored in RNAlater (Thermo Fisher Scientific; Waltham, MA, USA) at −20 °C.

### 4.4. RNA Sequencing Analysis

Total RNA (100 ng) derived from rat tongue exposed to 4NQO with or without BRB administration (5% or 10%) were used for total transcriptome library preparation using the NEBNext Ultra II Directional RNA Library Prep kit and rRNA Depletion kit (New England BioLabs; Ipswich, MA, USA). The fragment profile and the quantity of the resultant libraries were assessed by High Sensitivity DNA BioAnalyzer (Agilent; Santa Clara, CA, USA) and Qubit fluorometer (Thermo Fisher Scientific), respectively. Total transcriptome libraries were sequenced to a depth of 25–40 million clusters per sample using the HiSeq 4000 Paired-End 150 bp format.

### 4.5. Reverse Transcription Quantitative PCR

RT-qPCR was performed to validate the gene expression profiles obtained from RNA-Seq analysis. Total RNA extracted from rat tongue tissues using the AllPrep DNA/RNA kit (Qiagen; Valencia, CA, USA) was reverse transcribed using the High Capacity cDNA Reverse Transcription kit. (Applied Biosystems; Foster City, CA, USA). PCR amplification was performed using the PowerUp SYBR green master mix (Thermo Fisher Scientific). Primers were designed using the IDT real-time PCR design tool (Integrated DNA Technologies; Coralville, IA, USA). Primer sequences for the genes are shown in Table 4. Data were normalized to the reference gene *Bact* and gene expression was presented as fold induction over sentinel rats using the ddCt method.

### 4.6. Liquid Chromatography-Mass Spectrometry

Protein was precipitated from rat urine samples using cold methanol extraction. Urine–methanol solutions were allowed to incubate at 0 °C for 30 min before a 30 min centrifugation at 13,000× *g*. A total of 80 µL of the supernatant was transferred to a 2 µm microfiltration tube, followed by another centrifugation for 25 min at 13,000× *g*. The resulting solution was mixed in a 1:1 ratio with a 0.1% formic acid solution in distilled water, before transfer to an auto sampler vial for LC-MS analysis.

Liquid chromatography was carried out using the Agilent Dionex U3000 RSLC HPLC system with Zorbax SB–Ag columns, 3 × 150 mm, 3.5 µm (Thermo Fisher Scientific). Two solvents were prepared for use, solvent A (0.1% formic acid in distilled water) and solvent B (100% acetonitrile). Flow rate for chromatography was set at 250 µL/min, and a 55 min solvent gradient was run through the column with an injection volume of 1 µL. Mass spectrometry was performed on the resulting fractions using an LTQ-Orbitrap XL (Thermo Fisher Scientific) set to positive polarity with data dependent analysis (DDA) for the top 15 masses with a 15 s exclusion window, detecting masses ranging between 100–1200 *m*/*z*.

### 4.7. Nuclear Magnetic Resonance

A total of 180 μL of urine was mixed with 20 μL of 500 mM phosphate buffer in D_2_O, resulting in a pH of 7.4, together with 0.1 mM DSS (4,4-dimethyl-4-silapentane-1-sulfonic acid) for chemical shift referencing followed by transfer to a 3 mm NMR tube for NMR analysis. All NMR spectra were collected on a Bruker Advance III 850 MHz spectrometer equipped with a cryogenically cooled TCI probe at 298 K. The 2D ^13^C-^1^H HSQC spectra of all 15 urine samples (five samples of 4NQO exposed rats not receiving BRB treatment, five samples of 4NQO exposed rats treated with a 5% and 10% BRB diet) were collected with 512 × 2048 (N1 × N2) complex points along the two dimensions with 32 scans per increment. The spectral widths along the ^13^C and ^1^H dimensions were 34,211.06 and 10,204.08 Hz, respectively, and the transmitter frequency offsets were 75.00 and 4.70 ppm, respectively. The measurement time for each sample was 16 h. The data were zero filled two-fold along the ^13^C dimension, Fourier transformed, and phase- and baseline-corrected using NMRPipe [73].

### 4.8. Metabolite Data Extraction and Compound Identification

All NMR spectra of the urine samples were uploaded on the COLMAR web server for metabolite identification [4]. An in-house peak-picker and peak-fitting tool embedded in the COLMAR web server was used for peak picking and fitting. All spectra were referenced by internal standard DSS. For metabolite database query and matching, the ^1^H chemical shift cutoff was set to 0.03 ppm, the ^13^C chemical shift cutoff was set to 0.3 ppm, and the cutoff of the matching ratio (which is the ratio between the number of observed and expected cross-peaks for a given metabolite) was set to 0.6. The metabolite identification result was automatically generated by COLMAR and confirmed by manual inspection. A metabolite was classified as “identified” if it was found to be present in at least two samples of the same group. For quantitative analysis, all NMR spectra were normalized by the sum of the peak integrals (determined by peak fitting) of consensus peaks across all samples. Each metabolite fold change (BRB treated sample/non BRB treated sample) was calculated based on the average peak integral ratio of all peaks that were uniquely assigned to the metabolite. To minimize metabolite fold change uncertainties, all overlapped peaks, i.e., peaks assigned to multiple metabolites, were excluded for quantitative analysis [74,75].

### 4.9. Metabolite Data Extraction, Statistical Analysis and Compound Identification

All feature detection was performed using Progenesis QI with each run imported using the RAW XL MS data. Runs were aligned to the pooled QC samples (a combined set of all 20 samples), which were run every 10 samples. Sample alignment of the runs matched to pooled references below 85% were removed from statistical analysis and features of *p*-values less than 0.05 were considered statistically significant. Additionally, only those features which had MS/MS fragmentation analysis were considered as valid features. Each possible metabolite was tentatively identified using the Human Metabolome Database sdf file uploaded into Progenesis MetaScope with a precursor tolerance of 10 ppm and a theoretical fragmentation tolerance of 100 ppm along with a retention time window of 6 s. Adducts included for positive mode identification included M + H, M + Na, M + K, M + 2H, M + H − H2O, M + can + H, 2M + H, M + can + H, and M + 2Na − H. In total, 292 potential metabolic features were tentatively identified with HMDB codes and a 0.05 *p*-value cut-off.

### 4.10. Metabolic Pathway Analysis

The list of features from the Progenesis QI alignment and detection were submitted to MetabolAnalyst’s MS Peaks to Pathways module to determine the predicted pathway activity profiles (mummichog). For submission, the input included no *p*-value cutoff, along with the fold changes for each peak feature between groups. Analysis was performed on a 0.1 ppm mass error and 0.05 *p*-value cutoff with a total of 551 or 12.71% significant features with the Rattus Norvegicus KEGG pathway library.

### 4.11. Ingenuity Pathway Analysis

Metabolic and molecular interactions between differentially regulated metabolites and genes were explored using IPA (Qiagen). Each rat gene identifier was mapped to its corresponding gene in the Ingenuity Pathway Knowledge Base. Families of genes and metabolites that were upregulated or downregulated in BRB administered rats compared to the controls were integrated into predictive network models on the basis of interactions within a biological pathway as defined in the literature and contained in the IPA Knowledge Base.

### 4.12. RNA Statistical Analysis

RNA sequences were mapped to Rat Genome build Rnor_6.0 using tophat version 2 and then quantified using featureCounts software. Genes with raw read counts below five for more than 80% of samples within each condition were first filtered out. R package DEseq2 was used to normalize the data and identify genes differentially expressed between groups. The *p*-value cutoffs were determined by controlling the mean number of false positives. Heatmaps with hierarchical clustering were generated, and principal component analysis was performed.

RT-qPCR data were determined as means ± SEM, and statistical analyses were done by using Prism 5 software (GraphPad, La Jolla, CA, USA). An analysis of variance was used to model the gene expression data, and p values less than 0.05 were considered statistically significant.

## Figures and Tables

**Table 1:** Predicted pathway activity profiles based on the pathway analysis of LC-MS data from rat urine samples of 4NQO exposed rats fed control diet, or diet supplemented with 5% or 10% BRB as determined using Mummichog in MetaboAnalyst. Significant hits represent differentially expressed metabolites in the predicted pathway between rat groups.

Predicted Metabolic Pathways	Total Metabolite Hits	Significant Metabolite Hits
Steroid hormone biosynthesis	15	11
Glyoxylate and dicarboxylate metabolism	6	5
Vitamin B6 metabolism	5	4
Cysteine and methionine metabolism	9	5
Riboflavin metabolism	2	2
Tryptophan metabolism	11	5
One carbon pool by folate	6	3
Porphyrin and chlorophyll metabolism	9	4
Glycerophospholipid metabolism	7	3
Pyrimidine metabolism	13	5
Citrate cycle (TCA cycle)	4	2
Ascorbate and aldarate metabolism	5	2
Primary bile acid biosynthesis	5	2
Pantothenate and CoA biosynthesis	5	2
Purine metabolism	24	8
Folate biosynthesis	6	2
Inositol phosphate metabolism	7	2
Arginine and proline metabolism	18	5
Starch and sucrose metabolism	12	3
Pentose phosphate pathway	8	2
Tyrosine metabolism	13	3
Glutathione metabolism	9	2
Biosynthesis of unsaturated fatty acids	9	2
Glycolysis or Gluconeogenesis	10	2
Alanine, aspartate and glutamate metabolism	10	2
Fructose and mannose metabolism	11	2
beta-Alanine metabolism	11	2
Aminoacyl-tRNA biosynthesis	17	3
Galactose metabolism	19	3
Amino sugar and nucleotide sugar metabolism	20	3

**Table 2:** Unique and differentially expressed metabolites in the urine samples of 4NQO exposed rats fed the control diet, or diets supplemented with 5% or 10% BRB. Metabolites were identified by ‘Complex Mixture Analysis by NMR’ (COLMAR) followed by 2D ^13^C-^1^H HSQC NMR quantitative analysis. Metabolites with quantitative ratios listed were detected in at least two samples per group. Numbers represent the relative quantities of metabolites when compared to the 4NQO-exposed rat group that were fed a normal diet.

Metabolite Groups	Metabolite	Treatment Groups		
		Normal Diet	10% BRB Diet	5% BRB Diet
Unique Metabolites	4-Methyl-2-oxovaleric acid	*D	*ND	ND
	Delta-Hexanolactone	ND	D	ND
	Quinone	ND	D	ND
	Hydroquinone	ND	D	ND
	4-Aminohippuric acid	ND	ND	D
	Homovanillic acid	ND	D	D
	Hydroxyphenylacetylglycine	ND	D	D
	N-Acetylcysteine	ND	D	D
Downregulated Metabolites	alpha-D-Glucose-6-phosphate	1	0.7	0.48
	alpha-D-Glucose-1-phosphate	1	0.68	0.57
	N_methylnicotinamide	1	0.67	0.64
	Lactose	1	0.63	0.67
	Dihydrouracil	1	0.62	0.68
	Taurine	1	0.61	0.67
	D-Galactose	1	0.57	0.56
	D-Xylose	1	0.49	0.52
	Pyruvic acid	1	0.47	0.42
	Spermine	1	0.23	0.34
Upregulated Metabolites	Alpha-Hydroxyhippuric acid	1	5.25	1.86
	3-Methyl-oxopentanoic acid	1	3.73	1.54
	Chitosan	1	2.47	3.36
	Pipecolic acid	1	2.15	1.82
	N-Acetyl-D-glucosamine-phosphate	1	1.99	1.49
	3-Methoxy-4-Hydroxyphenylglycol-sulfate	1	1.6	1.84
	D-Glucuronic acid	1	1.59	1.54
	D-Sorbitol	1	1.59	1.78
	Myo-Inositol	1	1.57	1.51
	Stachyose	1	1.53	1.83
	DL-alpha-Glycerol-phosphate	1	1.35	1.4

*D (Detected in at least two samples of each group), *ND (Not detected in *any* of the five samples of each group).

**Table 3:** Differentially expressed PKA and AMPK pathway-associated genes in tongue samples of 4NQO exposed rats fed diets supplemented with 5% or 10% BRB as determined by RNA-Seq analysis. Data are expressed as fold change when compared to 4NQO exposed rats fed the control diet.

Gene	5% BRB		10% BRB		Expected Expression
	Expression	*p* Value	Expression	*p* Value	
*Cdc25a*	5.5	0.451	17.9	0.0304	Down
*Crebbp*	−1450.5	0.0114	−401.9	0.403	Up
*Gnas*	−1256.4	0.217	−3132.4	0.00908	Up
*Mapk13*	−29.0	0.141	−47.0	0.0279	Up
*Mppe1*	−1.3	0.281	−2.9	0.0307	Up
*Pde10a*	−11.0	0.0256	−9.0	0.0566	Up
*Pde4a*	−60.9	0.0748	−75.7	0.0336	Up
*Phkg1*	−9.1	0.696	−53.5	0.0417	Up
*Pik3c2g*	−3.7	0.00638	-3.7	0.00638	Up
*Ppp1r14c*	16.0	0.0712	20.6	0.0272	Down
*Prkaa2*	−136.8	0.0409	−149.2	0.0287	Up
*Prkar1a*	−79.5	0.469	−270.1	0.0303	Up
*Ptpre*	7.7	0.00969	5.5	0.0443	Down
*Smad4*	−479.6	0.000238	−367.4	0.0015	Up
*Tgfbr2*	−82.4	0.113	−123.0	0.0278	Up
*Ywhag*	−624.7	0.033	−628.7	0.0321	Up

**Table 4:** Primer sequences used for RT-qPCR analysis of glycolytic pathway genes.

Gene	Forward Primer	Reverse Primer
*Aldoa*	CCCCAAGTTATCAAGTCCAAGG	GTCGGCTCCATCCTTCTTATAC
*Aldob*	TCAAGGAGAAGGGAATTGTGG	TTCTTGTACTGAGCACAGCG
*Aldoc*	CCTGTCCCATCAAATATAGCCC	TGGCATTGAGGTTGAGTGATG
*Gnpda1*	TCACACCCTCTTCAATGGC	GATTCTTTGATGGTGATCTTGCC
*Hk1*	ACACCCATTGTCACCGAAG	AAGAACCCACTTGCGAAATTG
*Hk2*	GCTGTGAAAATGTTGCCTACC	CATTGTCCGTCACCCTTACTC
*Hk3*	AGTGGAGCTGAATGTGGTTG	TCTTCCATATAGCAGGCGTTG
*Gck*	GCCACAATGATCTCCTGCTAC	TCCCTCATCCCCTTCCAC
*Gpi*	AGGTGCTGGACAAGATGAAG	GAGAGCTTCAGTCACCATGAG
*Pfkm*	TGGTACGAGTTGGCATCTTC	ACTTCCAATCACCGTGCC
*Pfkl*	GGAAAGCCTATCTCATCCAGC	CCATACCCATCTTGCTACTCAG
*Pfkp*	TCACTGAGAGGAAATCAAGCTG	TGTAGGTGTTCAGGTTGCC
*Tpi1*	AGATAATGTGAAGGACTGGTGC	CAGAGACGTTGCATTTCAGC
*Gapdh*	TGGTTACAATACAGTTACAGACTAGG	GTGGTTGTAAATGGAAGAAACATCT
*Pgk1*	TGACTTTGGACAAGCTGGAC	CAGAATTTGATGCTTGGGACAG
*Pgam2*	CCTAAGATTAAGGCTGGCAAGAG	GGTTCAGCTCATAGACAATGGG
*Eno1*	CCGAGACAATGATAAGACCCG	TCTCCTGCTCCACAACATTC
*Eno2*	TCAAGGACAAGTACGGCAAG	CATACCAATCACCATCTTTTCCG
*Eno3*	GAATCCCGACCTTGTACTTCC	GCCAACTTGTTTCCAGCATG
*Pkm2*	GTGGAGATGCTGAAGGAGATG	AGGTCGGTAGAGAATGGGATC
*Pklr*	CACCTCTGCCTTCTGGATATC	TCGTGCAATGTTCATCCCTG
*Bact*	CACTTTCTACAATGAGCTGCG	CTGGATGGCTACGTACATGG
